# Supplementary material for: P-TRAP: a Panicle Trait Phenotyping tool
Source: BMC Plant Biol. 2013 Aug 29;13:122. doi: 10.1186/1471-2229-13-122 (PMC3848748; doi:10.1186/1471-2229-13-122)
Supplement: Additional file 7 — P-TRAP source code. The Netbeans project that contains the source code of the application. [file 1471-2229-13-122-S7.zip › javadoc.zip!/javadoc/org-bioalg-widgetfactory/org/bioalg/widgetfactory/CircleWidget.html]

CircleWidget (WidgetFacory)


JavaScript is disabled on your browser.


- Package
- Class
- Tree
- Deprecated
- Index
- Help

*org.bioalg.widgetfactory 1.0*

- Prev Class
- Next Class

- Frames
- No Frames

- All Classes

- Summary:
- Nested |
- Field |
- Constr |
- Method

- Detail:
- Field |
- Constr |
- Method


org.bioalg.widgetfactory

## Class CircleWidget

- java.lang.Object
- - org.netbeans.api.visual.widget.Widget
  - - org.bioalg.widgetfactory.CircleWidget

- All Implemented Interfaces:
  :   javax.accessibility.Accessible, org.openide.util.Lookup.Provider

  ---

    

  ```
  public class CircleWidget
  extends org.netbeans.api.visual.widget.Widget
  ```

  Create a circle widget to use used in the graph editor.

  Author:
  :   Faroq AL-Tam based on an example provided by Sun for the use of
      visual liberary

- - ### Nested Class Summary

    - ### Nested classes/interfaces inherited from class org.netbeans.api.visual.widget.Widget

      `org.netbeans.api.visual.widget.Widget.Dependency`
  - ### Constructor Summary

    Constructors

    Constructor and Description

    `CircleWidget(org.netbeans.api.visual.widget.Scene scene,
    double radius,
    java.awt.Color color)`

    The constructor function.
  - ### Method Summary

    Methods

    | Modifier and Type | Method and Description |
    | --- | --- |
    | `void` | `attachLabel(org.netbeans.api.visual.widget.Scene scene, java.lang.String string)` Add a label the widget |
    | `protected java.awt.Rectangle` | `calculateClientArea()` |
    | `java.awt.Color` | `getColor()` |
    | `double` | `getRadius()` |
    | `boolean` | `isfixed()` |
    | `protected void` | `paintWidget()` |
    | `void` | `setColor(java.awt.Color color)` |
    | `void` | `setIsfixed(boolean isfixed)` |
    | `void` | `setRadius(double radius)` |

    - ### Methods inherited from class org.netbeans.api.visual.widget.Widget

      `addChild, addChild, addChild, addChild, addChildren, addDependency, bringToBack, bringToFront, convertLocalToScene, convertLocalToScene, convertSceneToLocal, convertSceneToLocal, createActions, equals, getAccessibleContext, getActions, getActions, getBackground, getBorder, getBounds, getChildConstraint, getChildren, getClientArea, getCursor, getCursorAt, getDependencies, getFont, getForeground, getGraphics, getLayout, getLocation, getLookup, getMaximumSize, getMinimumSize, getParentWidget, getPreferredBounds, getPreferredLocation, getPreferredSize, getResourceTable, getScene, getState, getToolTipText, hashCode, isCheckClipping, isEnabled, isHitAt, isOpaque, isPreferredBoundsSet, isRepaintRequiredForRevalidating, isValidated, isVisible, notifyAdded, notifyBackgroundChanged, notifyFontChanged, notifyForegroundChanged, notifyRemoved, notifyStateChanged, paint, paintBackground, paintBorder, paintChildren, removeChild, removeChildren, removeChildren, removeDependency, removeFromParent, repaint, resolveBounds, revalidate, revalidate, setAccessibleContext, setBackground, setBackgroundFromResource, setBorder, setBorder, setCheckClipping, setChildConstraint, setCursor, setEnabled, setFont, setFontFromResource, setForeground, setForegroundFromResource, setLayout, setMaximumSize, setMinimumSize, setOpaque, setPreferredBounds, setPreferredLocation, setPreferredSize, setResourceTable, setState, setToolTipText, setVisible, updateResources`
    - ### Methods inherited from class java.lang.Object

      `clone, finalize, getClass, notify, notifyAll, toString, wait, wait, wait`

- - ### Constructor Detail


    - #### CircleWidget

      ```
      public CircleWidget(org.netbeans.api.visual.widget.Scene scene,
                  double radius,
                  java.awt.Color color)
      ```

      The constructor function.

      Parameters:
      :   `scene` - A `Scene` object where this widget will be used in.
      :   `radius` - double, the circle radius.
      :   `color` - The `Color` of the circle.
  - ### Method Detail


    - #### isfixed

      ```
      public boolean isfixed()
      ```


    - #### setIsfixed

      ```
      public void setIsfixed(boolean isfixed)
      ```


    - #### calculateClientArea

      ```
      protected java.awt.Rectangle calculateClientArea()
      ```

      **Overrides:**
      :   `calculateClientArea` in class `org.netbeans.api.visual.widget.Widget`


    - #### paintWidget

      ```
      protected void paintWidget()
      ```

      **Overrides:**
      :   `paintWidget` in class `org.netbeans.api.visual.widget.Widget`


    - #### attachLabel

      ```
      public void attachLabel(org.netbeans.api.visual.widget.Scene scene,
                     java.lang.String string)
      ```

      Add a label the widget

      Parameters:
      :   `scene` - the `Scene` where this label will be used in.
      :   `string` - The label's string.


    - #### getColor

      ```
      public java.awt.Color getColor()
      ```


    - #### setColor

      ```
      public void setColor(java.awt.Color color)
      ```


    - #### getRadius

      ```
      public double getRadius()
      ```


    - #### setRadius

      ```
      public void setRadius(double radius)
      ```


- Package
- Class
- Tree
- Deprecated
- Index
- Help

*org.bioalg.widgetfactory 1.0*

- Prev Class
- Next Class

- Frames
- No Frames

- All Classes

- Summary:
- Nested |
- Field |
- Constr |
- Method

- Detail:
- Field |
- Constr |
- Method
